# Expression of concern: Heterostructured nanocomposite tin phthalocyanine@mesoporous ceria (SnPc@CeO_2_) for photoreduction of CO_2_ in visible light

**DOI:** 10.1039/d4ra90151g

**Published:** 2025-01-17

**Authors:** Pawan Kumar, Arvind Kumar, Chetan Joshi, Raghuvir Singh, Sandeep Saran, Suman L. Jain

**Affiliations:** a Chemical Sciences Division, CSIR-Indian Institute of Petroleum Dehradun 248005 India suman@iip.res.in; b Analytical Science Division, CSIR-Indian Institute of Petroleum Dehradun 248005 India

## Abstract

Expression of concern for ‘Heterostructured nanocomposite tin phthalocyanine@mesoporous ceria (SnPc@CeO_2_) for photoreduction of CO_2_ in visible light’ by Pawan Kumar *et al.*, *RSC Adv.*, 2015, **5**, 42414–42421, https://doi.org/10.1039/C5RA06449J.

*RSC Advances* is publishing this expression of concern in order to alert our readers to the fact that concerns have been raised regarding the reliability of the XRD data in Fig. 4. An investigation is underway, and an expression of concern will continue to be associated with this article until a final outcome is reached.

Signed: Laura Fisher, Executive Editor, *RSC Advances*

Date: 12th December 2024

